# Mind the Gap: Tools for the Diagnosis and Assessment of Orthorexia Nervosa Based on the Recent Consensus Definition. Reply to Meule, A. Comment on “Sifakaki et al. Orthorexia Nervosa Practices in Rheumatoid Arthritis: The DORA Study. *Nutrients* 2023, *15*, 713”

**DOI:** 10.3390/nu15081985

**Published:** 2023-04-20

**Authors:** Maria G. Grammatikopoulou, Konstantinos Gkiouras, Georgios Marakis, Maria Sifakaki, Anastasia Petropoulou, Lorenzo M. Donini, Helen M. Lindqvist, Dimitrios P. Bogdanos

**Affiliations:** 1 Unit of Immunonutrition and Clinical Nutrition, Department of Rheumatology and Clinical Immunology, Faculty of Medicine, School of Health Sciences, University of Thessaly, Biopolis, GR-41110 Larissa, Greece; 2 Nutrition and Food Standards Unit, Hellenic Food Authority, 124 Kifisias Avenue & Iatridou 2, GR-11526 Athens, Greece; 3 Department of Experimental Medicine, Sapienza University, 00185 Rome, Italy; 4 Department of Internal Medicine and Clinical nutrition, Institute of Medicine, Sahlgrenska Academy, University of Gothenburg, 40530 Gothenburg, Sweden

In a recent manuscript, our team published the results of an original pilot cross-sectional study assessing orthorexia nervosa (ON) tendencies among patients with rheumatoid arthritis (RA) [1]. The study revealed the existence of ON tendencies among patients with an RA diagnosis, with greater tendencies being associated with the female gender and reduced ON tendencies with increasing age and body mass index [1]. The main conclusions of this study were that (i) health professionals must become familiar with the problem of ON, (ii) patient screening is important, (iii) more research is required to evaluate the degree of ON tendencies among patients with RA, (iv) ideally, with a prospective study design, in order to untangle the consequences of ON in distinct disease outcomes and prognoses [1]. Overall, due to the study’s limitations which were extensively detailed in the manuscript, namely the relatively small sample size, the cross-sectional design, the lack of a control arm, the patient-reported outcomes, and the use of the only tool that has been translated and validated in the Greek language to date (the ORTO-15), caution was taken to present the results without pompous conclusions and extrapolations [1]. 

Meule [2] commented that our manuscript concluded that “patients with RA who receive medical nutrition therapy (MNT) show orthorexic tendencies”, which unfortunately consists of an inaccurate and misleading extrapolation of our work and an inexistent conclusion. In fact, our conclusions suggest that “the promotion of healthy eating is of central importance in chronic diseases, including RA, (however) it must be delivered by experts…” [1]. The core of the comment made was that “doubts (existed) about the conclusion that patients with RA who receive MNT show orthorexic tendencies”. The answer is that unfortunately we do not know and are unable to answer, as this was not the hypothesis of our study. Our study did not compare patients with RA receiving MNT against those following their usual diet, so this conclusion was not only irrelevant to our work and missing from our manuscript but also impossible to make, as MNT was not even recorded.

In our study [1], the ORTO-15 [3,4] was used to evaluate ON tendencies. The ORTO-15 was first introduced in the year 2005 [3], and since then, the original version or variations of the tool have been translated into dozens of languages. Meule criticized the use of this specific tool and stressed the fact that research has been advised against the use of ORTO-15 [2]. We greatly appreciate the comment, which was also clearly listed within the limitations of our study as follows “…An additional limitation of the study involves the use of the ORTO-15 tool, which has been previously criticized by some researchers”. As we have previously noted [1,5,6,7], although a variety of tools have been proposed for the assessment of ON, all of them carry limitations, with the most important one being that they all preceded the recently published consensus definition and diagnostic criteria of ON [8]. In this manner, all of the existing tools evaluate some, but not all clinical aspects and characteristics of ON. In parallel, despite its inherited limitations that have been discussed in the literature and acknowledged in our work, the ORTO-15 and its variations consist of the most widely used tools to date for the assessment of ON [9], covering 62% of the PubMed hits: more than all of the remaining questionnaires combined (Figure 1). Furthermore, the ORTO-15 and its variations consist of the sole validated, translated and culturally adapted tools in Greece today [4].

Nonetheless, as stated in the consensus for the definition of ON [8], since the clinical aspects of ON have now been defined, all existing tools should be re-evaluated and revised, and new, more accurate tools must be developed for screening and identifying relevant patients and investigating the prevalence of ON. Again, this is something that was clearly communicated in our manuscript [1], while the development of novel tools tailored to the definition of ON is anticipated by all researchers working within the ON bubble.

ON is a rather new but important disorder, attracting great interest from the scientific community. Given the recent work on the definition of ON, we all need to be cautious when proposing pre-existent questionnaires since none of these can grasp the extent of ON, as defined in the recent consensus. In simple wording, all existing tools to date are equally ineffective in covering all ON aspects and clinical signs of the disorder, irrespective of confirmatory factor analyses, validation, consistency or reliability measures. Until a novel tool for ON diagnosis can be developed based on the recent consensus, researchers working on ON inevitably have to utilize one of the already existing and ineffective tools and report their limitations. With this communication, we hope we have once again clarified the conclusions of our work in anticipation of the development of new ON-specific tools, stemming from the consensus report.

## Figures and Tables

**Figure 1:**
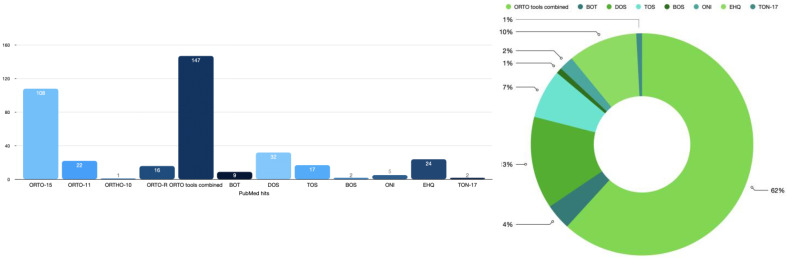
Absolute number (left) and percent (%, right) of the PubMed hits regarding the tools used for the assessment of ON (Accessed on 3 March 2023). BOS: Barcelona Orthorexia Scale; BOT: Bratman Orthorexia Test; DOS: Düsseldorf Orthorexia Scale; EHQ: Eating Habits Questionnaire; ON: Orthorexia nervosa; ONI: Orthorexia nervosa inventory; ORTO combined: includes the ORTO-15, ORTO-11, ORTHO-10, and the ORTO-R; TOS: Teruel Orthorexia Scale; TON-17: Test of Orthorexia Nervosa.
